# Tracheotomy in Croup

**Published:** 1875-06

**Authors:** W. C. Phelps


ART. II. *Tracheotomy in Croup—Successful Result in a Case where Diagnosis was Unmistakable.* By W. C. PHELPS, M. D.

Successful tracheotomy in a case where the membranous character of the disease is demonstrated by ejection of a false membrane is of interest to all physicians since the fatality attending tracheotomy in croup has led many experienced and observing physicians to believe that though the successful cases of tracheotomy were sufficiently common, yet that the success was due to the fact that the membranous character was wanting, and that the cases would have recovered without tracheotomy, in other words, that tracheotomy would not of itself prove fatal, or greatly endanger life—would not prevent natural recovery. The following case has some important bearing upon all the points connected with tracheotomy in croup, and is reported because it so forcibly demonstrates, the now very generally accepted idea that *tracheotomy is proper to be recommended in membranous croup when it becomes evident that recovery is highly improbable without it.* Authors properly caution against too long delay and most certainly there is great danger from delay. Positive diagnosis is not always obtainable, and the careful physician watches the condition and progress of the case, thus requiring often valuable time. It is far better to operate upon a case which might possibly recover without it, then to delay operation until death is inevitable in cases certainly fatal unless operated upon. The facts of the following case are sufficiently suggestive to the experienced, thoughtful physician and need little comment:

On the morning of May 10th, I was called in the absence of Dr. M. G. Potter, the attending physician, to the Buffalo Orphan Asylum, to see S. C., aged six years, who had been taken sick during the preceding night, and the symptoms present being different from those of spasmodic croup, awakened in the mind of the experienced matron, Mrs. McPherson, a suspicion that the child had membranous croup. I will state in this connection that four weeks before this a bright little girl of the same age, and of a good constitution, had died of membranous croup after an illness of three days duration, and from the beginning of March to the present time, June 15th, there have been about twenty cases of spasmodic croup. It is very unusual in the history of the institution that there should be such a large number of cases of disease of any form, as during the ten years intervening between 1864 and 1874, there was but very little sickness and only one death. A thorough cleaning of the house and premises has been ordered, with a view to the prevention of this epidemic of croup.

I found the patient with a pulse of 120, temperature 102° F., tongue with a white cream-like coating, respiration 30 per minute, absence of voice, considerable difficulty in breathing, and the characteristic cough of croup, percussion over the chest normal, but auscultation showed sonorus and sibilant rales over both lungs. Inspection of the throat showed a general conjestion of the pharynx, but without much swelling, and the absence of any deposit. The diagnosis was membraneous croup, and the following prescription made:

℞ Calomel grs. vii.

Pulv. James, grs. vii.

Pulv. Dovers, grs. xv., M. } Ft Pulv. No. xv.

Give one powder every fourth hour; also two grains of quinine every second hour. The temperature of the room to be kept at 75° F., and the air moistened by water vaporized. Inhalations of steam from hot water poured on unslacked lime were given every second hour; the bowels kept open, warm applications to the throat, and an emetic of ipecacuanha night and morning during the first two days of treatment. On the afternoon of the 12th inst., the third day of treatment, I called Drs. Miner and Rochester in consultation. At this time the condition of the patient was as follows: Pulse 120, tongue thickly coated, and somewhat dry. considerable difficulty in breathing, the condition of the throat unchanged. The nurse informed us that at times the child would be taken with what she called sinking spells, and that at such times the pulse would become weak and very frequent with increased embarrassment of respiration. The treatment above detailed was continued with the addition of stimulants, with the understanding that if the breathing became more difficult, tracheotomy would be indicated.

On the morning of the next day, the 13th inst., the patient was every way worse; the nurses reported that they had been unable to give either medicine or nourishment by the mouth since 12 o'clock midnight, and had ceased attempting to give anything, thinking it better to let the child die as easily as possible. While I was examining into his condition he was suddenly taken with a severe paroxysm of choking, which was somewhat relieved by the coughing up of a false membrane, about two inches in length, a complete cylinder, shaped to the interior of the trachea below the larynx.

Our diagnosis was now confirmed, but the respiration was still very difficult, more difficult in fact than before the expulsion of the membranous cast, and at the request of the father of the child and the matron of the asylum, I made preparations for the performance of tracheotomy, Drs. Miner and Rochester again very kindly met me in consultation, and the propriety of the operation was concurred in. At my request Dr. Miner proceeded to operate. Drs. W. W. Miner, Brush and Barnes assisting; the anesthetic being administered by Dr. Rochester. The operation was successfully made in the usual manner, below the isthmus of the thyroid gland, and after a large amount of mucous was expelled through the opening, a curved double tube was passed into the trachea and secured by tapes. The patient now breathed very freely through the tube, the external opening of which was covered with gauze, and after the effect of the anesthetic had passed off, was removed from the operating table to his bed. From this time forward in the treatment of the case, all medicines were withheld, excepting a Dover's powder at night, and the diet consisted exclusively of milk. The inner tube was removed and cleaned frequently on account of the large quantity of mucous which was present in the lungs and coughed out through it. The general condition of the child began at once to improve, and on the eighth day the tube was removed from the trachea, the opening being left open to close by granulation. On the fourteenth day after the operation the wound in the trachea was closed, so that respiration was naturally and easily performed, and the voice sounds were perfect. The patient was discharged cured.

